# Open pipelines for integrated tumor genome profiles reveal differences between pancreatic cancer tumors and cell lines

**DOI:** 10.1002/cam4.360

**Published:** 2015-01-04

**Authors:** Jeremy Goecks, Bassel F El-Rayes, Shishir K Maithel, H Jean Khoury, James Taylor, Michael R Rossi

**Affiliations:** 1 Computational Biology Institute, George Washington UniversityAshburn, Virginia, 20147; 2 Department of Hematology and Medical Oncology, Emory UniversityAtlanta, Georgia; 3 Department of Surgery, Division of Surgical OncologyEmory University, Atlanta, Georgia; 4 Department of Biology, Johns Hopkins UniversityBaltimore, Maryland; 5 Department of Radiation Oncology, Division of Cancer Biology, Emory UniversityAtlanta, Georgia

**Keywords:** Analysis pipelines, bioinformatics, galaxy, genomic tumor profiles, pancreatic cancer

## Abstract

We describe open, reproducible pipelines that create an integrated genomic profile of a cancer and use the profile to find mutations associated with disease and potentially useful drugs. These pipelines analyze high-throughput cancer exome and transcriptome sequence data together with public databases to find relevant mutations and drugs. The three pipelines that we have developed are: (1) an exome analysis pipeline, which uses whole or targeted tumor exome sequence data to produce a list of putative variants (no matched normal data are needed); (2) a transcriptome analysis pipeline that processes whole tumor transcriptome sequence (RNA-seq) data to compute gene expression and find potential gene fusions; and (3) an integrated variant analysis pipeline that uses the tumor variants from the exome pipeline and tumor gene expression from the transcriptome pipeline to identify deleterious and druggable mutations in all genes and in highly expressed genes. These pipelines are integrated into the popular Web platform Galaxy at http://usegalaxy.org/cancer to make them accessible and reproducible, thereby providing an approach for doing standardized, distributed analyses in clinical studies. We have used our pipeline to identify similarities and differences between pancreatic adenocarcinoma cancer cell lines and primary tumors.

## Background

A promising path toward personalizing cancer treatment is using genomic features of tumors to guide treatment. Tumor features such as gene mutations 1,2, differential gene expression 3,4, and structural variation 5,6 have proven useful in predicting and personalizing cancer treatment. For the majority of tumors, though, finding a single feature that leads to a definitive treatment with durable response has been elusive. Therefore, developing effective treatments for many tumor types requires multiple targeted approaches informed by comprehensive tumor profiles merged with public and private patient data to identify precise targets 7.

Comprehensive genomic profiles of tumors derived from high-throughput sequencing data holds significant promise for better understanding the biology which drives their growth and resistance to standard therapies 8,9. New information can be derived by combining data from multiple characteristics. For instance, mutations in overexpressed genes can be found by combining mutations from exome resequencing with gene expression computed from transcriptome sequencing. Activating mutations in genes that drive growth and proliferation are often promising drug targets and many current cancer therapies have been based on the concept of oncogene addiction 10–12.

For cancers with poor outcomes, using a multi-faceted tumor profile to identify better targeted agents or combinations of drugs is required. Large public databases that include cancer genome information such as COSMIC 13, the Drug–Gene Interaction (DGI) Database 14, and the Cancer Cell Line Encyclopedia 15, are making this task more feasible. Current approaches match a known genomic aberration to a known drug, such as the *BRAF* p.V600E mutation and vemurafenib 16,17, but there is an increasing need to test combinations of drugs in clinical trials. However, many of these trials require preclinical models for evidence of efficacy, and most of these models currently fail to account for multiple somatic events that contribute to therapeutic response. Because the use of cell lines for personalized oncology appears to be a more cost effective approach than xenograph models 18, we have chosen to develop a tool that aligns individual tumor data with available cell lines in an effort to help accelerate precision investigation of preclinical models of therapeutic response.

Realizing an approach to personalized oncology that creates an integrated genomic profile of a tumor and then uses the profile together with large public databases is a challenging endeavor that requires pipelines with many steps and tools. Ensuring that these pipelines and their output are accessible to research-clinicians, especially those with limited computational skills, is critical. It is also important that these pipelines yield reproducible analyses so that their results can be used and also serve as a foundation for future work 19. For these reasons, a pipeline/workflow platform is ideal. Pipeline platforms such as GenePattern 20, Taverna 21, and Synapse 22 have been used for cancer genomics but, to the best of our knowledge, not for personalized oncology.

We have developed three pipelines for personal oncology and integrated them into Galaxy, a popular Web-based genomic workbench that supports pipelines 23–26. Collectively, these pipelines—an exome analysis pipeline, a transcriptome (RNA-seq) analysis pipeline, and an integrated variant analysis pipeline—analyze a tumor sample to identify rare and deleterious mutations, druggable mutations, and drugs which may be effective for a tumor. Integrating the pipelines into Galaxy makes the pipelines and the data produced from them widely accessible, reproducible, and sharable. Galaxy's Web interface ensures accessibility and reproducibility of the pipelines for a wide audience, especially those with limited programming skills. Galaxy's collaboration framework provides a channel for widely sharing the pipelines and ensuring that others can easily use them. Together, Galaxy and the pipelines facilitate standardization of a data analysis platform that can run locally with appropriate securities but the analyses can be easily shared and collated across sites in multicenter clinical trials.

We have validated our pipelines by analyzing high-throughput sequencing data from three well-characterized pancreatic cancer cell lines. Finally, we have used the pipelines to identify mutational similarities and differences between the cell lines and six primary pancreatic adenocarcinoma (PAC) tumors.

## Implementation

**Figure 1:**
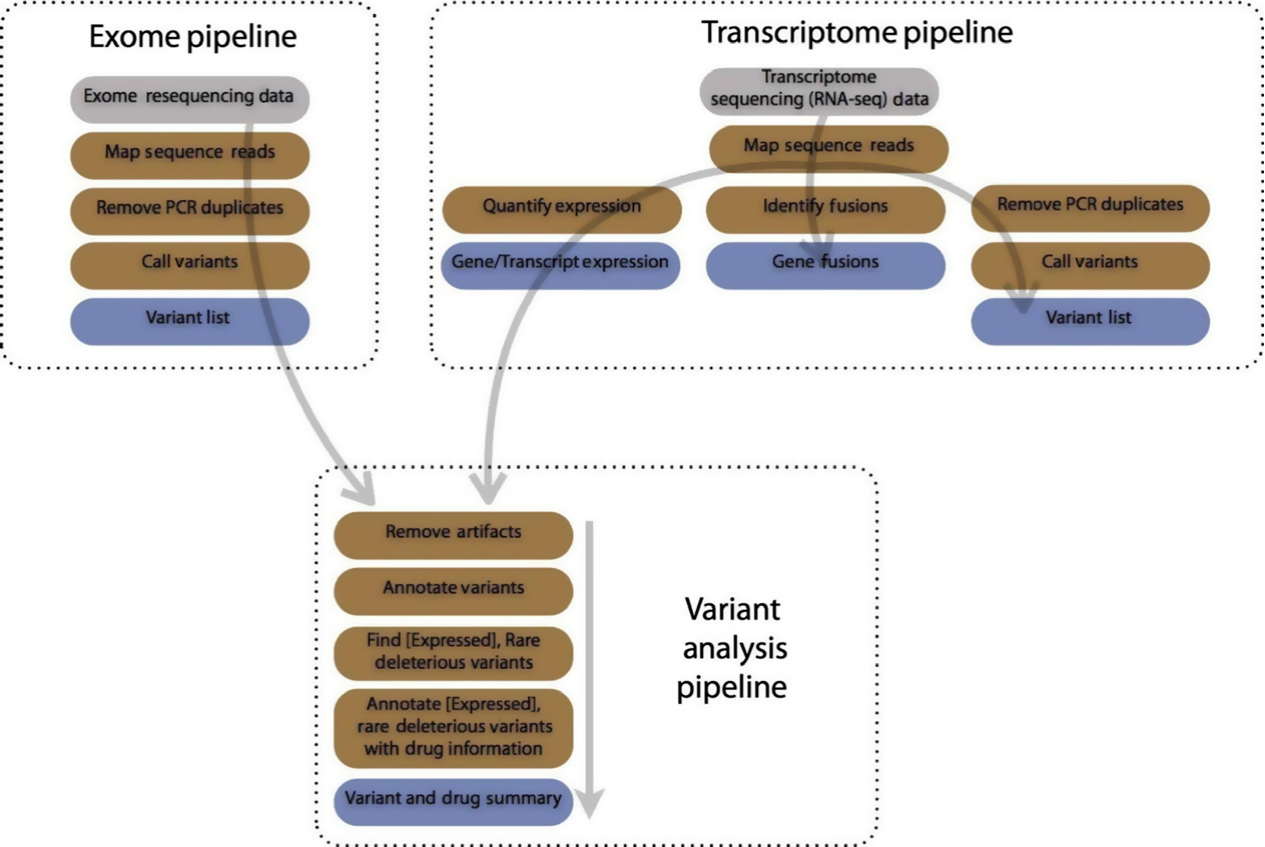
Diagram of available pipelines; inputs are gray, steps are brown, and outputs are blue. The *exome pipeline* processes high-throughput resequencing data, whether from targeted or whole exome, and produces a list of variants. The *transcriptome pipeline* processes high-throughput transcriptome sequencing data to produce gene and transcript expression, potential gene fusions, and a list of variants. The *variant analysis pipeline* annotates variants from either the exome or transcriptome pipeline to identify rare, deleterious and druggable (RDD) variants and RDD variants that occur in highly expressed genes. Variant annotations include functional impact, allele frequency (from 1000 Genomes and the Exome Sequencing Project), dbSNP id, COSMIC information, and additional information from a variety of public sources. Druggable variants are annotated with information about their drug interactions. Output from this pipeline is a summary of RDD variants and known drug interactions. If gene expression information is provided from the transcriptome pipeline, rare and deleterious variants as well as druggable variants and associated drugs are provided for highly expressed genes. Variants in highly expressed genes are often promising drug targets.

We have created three general pipelines that work together (Fig.1):

- An exome processing pipeline analyzes whole or targeted tumor exome resequencing data and identifies small variants (SNPs and indels).
- A whole transcriptome (RNA-seq) processing pipeline analyzes tumor RNA-seq data (a) to find small variants and gene fusions and (b) computes gene expression.
- An integrated variant analysis pipeline that processes variants from the exome or transcriptome pipelines, together with public databases, to identify (i) rare and deleterious (RD) variants; (ii) druggable RD variants and associated drugs. When gene expression data are available from the transcriptome pipeline, an integrated analysis is performed to identify (iii) RD variants in highly expressed genes; (iv) druggable RD variants in highly expressed genes and associated drugs.

The integrated analysis in the final pipeline focuses on variants in highly expressed mutant transcripts likely to be druggable targets. The tools chosen for these pipelines are widely used and well maintained, ensuring that they perform well on a variety of different data. However, alternative tools can also be incorporated to these pipelines as required.

### Exome pipeline

This pipeline generates a list of small variants (SNPs, insertions, and deletions) from either whole or targeted tumor exome resequencing data. In order, reads are mapped using BWA 27, PCR duplicates are removed using Picard (http://picard.sourceforge.net/), and variants are called using VarScan2 28. This approach—mapping reads, remove duplicates, and calling variants—is well established for obtaining variants from exome data. No matched normal exome sequencing data are required to run this pipeline.

### Transcriptome (RNA-seq) pipeline

This pipeline uses RNA-seq data to characterize a tumor in three ways: small variants, gene fusions, and gene expression. The first step in the pipeline is mapping RNA-seq reads using Tophat2 29. Obtaining small variants from mapped reads is done the same way as it is in exomes: PCR duplicates are removed and variants are called. This approach for calling variants from RNA-seq has been validated previously 30. The pipeline uses the popular Tophat-Cufflinks protocol 31 produce gene expression results and Tophat-Fusion 32 to detect potential fusions.

### Integrated variant analysis pipeline

This pipeline analyzes variants from the exome or transcriptome pipeline to identify rare, deleterious (RD) variants and also druggable RD variants together with associated drugs. When transcriptome data are available, variants and drugs in highly expressed genes are computed as well.

The first step in this pipeline is removing variants resulting from sequencing errors, and this is done by removing variants with a low allele frequency. The minimum required allele frequency can be set when the workflow is run. We found that using a minimum allele frequency of 10% worked well for variants called from exome sequencing of a homogenous population of cells, such as a cultured cell line. Conversely, for variants called from transcriptome sequencing of a tumor, which has a mixed population of cells, we found that an allele frequency of 30% worked well.

Next, ANNOVAR 33 is used to annotate variants with mutation type (e.g. synonymous, stop-gain, frameshift), allele frequencies in common public databases such as 1000 Genomes 34 and the Exome Sequencing Project 35, and COSMIC 13 annotations. To obtain rare and deleterious variants, variants with a minor allele frequency greater than 0.01% in either 1000 Genomes and the Exome Sequencing Project data are removed and only nonsynonymous mutations, frameshifts, and stop-gain/losses are kept. Additional annotations can be added and more aggressive annotation filtering can be applied as required by changing the filtering criteria.

Next, the rare and deleterious mutations are annotated using the DGI database, a meta-database that includes gene–drug interaction results from many expert-curated and automatically generated drug databases 14. For each gene that has a rare and deleterious mutation, the DGI database provides a list of drugs that are thought to be effective, along with the database source of the interaction and, if available, interaction type (e.g., inhibitor). By default, only expert-curated results are included in the pipeline's results.

The final outputs of the pipeline are (a) a list of rare and deleterious mutations; (b) a list of druggable rare and deleterious mutations; and (c) a list of potential drugs associated with the druggable mutations. When gene expression data are available from the transcriptome analysis pipeline, variants are annotated with the expression level for the gene where they occur. Then, variants with expression greater than 10 FPKM are labeled as highly expressed and the pipeline identifies rare, deleterious variants in highly expressed genes, the subset of these variants that are druggable, and associated drugs. As noted earlier, variants in expressed genes and drugs targeting variants in expressed genes may be especially interesting to clinician-researchers. The goal is to use these lists of rare, deleterious and druggable mutations to in tumors to direct rational preclinical testing of combination therapies in cell lines and eventually treatment of patients.

### Pipeline separation

Variant analysis and interpretation is a challenging part of using molecular profiles to personalize cancer treatment, and we anticipate that investigators may try experimenting with and adapting the variant analysis workflow to meet their needs. The separation in our pipelines affords experimentation with the variant analysis workflow. The exome and transcriptome analysis pipelines perform operations that are self-contained, resource-intensive, and slow. Read mapping, variant calling, and quantifying gene expression are most resource-intensive steps, each requiring two or more hours on typical computing clusters to complete. On the other hand, the variant analysis pipeline integrates the outputs of the exome and transcriptome pipeline with public databases, requires few resources, and is very fast. Using a personal computer, the whole pipeline typically finishes in less than 30 min. By placing the slow steps in the exome and transcriptome analysis pipelines, it is fast and easy to experiment with the variant analysis workflow, such as by changing the allele frequency or the databases used.

### Advantages of galaxy integration

We have implemented these pipelines as workflows in Galaxy (http://galaxyproject.org), a Web-based workbench for doing genomic analyses. Galaxy integration offers many benefits for investigators using these pipelines.

Galaxy can be accessed in a variety of different ways, depending on an investigator's bioinformatics skills. Investigators with limited bioinformatics experience can use Galaxy via a graphical Web-based interface for running workflows and visualizing and sharing data. Only a Web browser is required to use all of Galaxy's features and a novice bioinformatician can easily upload FASTQ or BAM files, execute workflows, and generate summary tables and graphics. For investigators with significant bioinformatics experience, Galaxy's API can be used to run workflows from scripts. Using the Galaxy API and Bioblend 36, we developed Python scripts to automatically execute the pipelines on sequencing data from numerous pancreatic cancer samples, the results of which we discuss in detail below. We used Galaxy's Web interface to experiment with different settings for our workflows, visualize results, and share our workflows and data.

Galaxy records the inputs and parameters used for workflows and tools, so every pipeline run is recorded and reproducible. Data produced from our pipelines can be visualized in Galaxy's visual analysis framework 37,38. Investigators can visualize the very large data sets produced by the pipelines in their Web browser using a genome browser, Circos plot 39, and other visualizations. Investigators can also experiment with and visualize tool output using different parameter values in order to choose parameters best suited to their analyses.

We have used Galaxy's sharing features to make our pipelines widely available. We created a Galaxy Page (http://usegalaxy.org/cancer) as an online, interactive supplement for this work. The page briefly describes the workflows, and embedded in the page are the workflows themselves, analysis histories generated from the pipelines using cancer cell line data, and visualizations of data generated from the pipeline. From the page, investigators can copy embedded histories, workflows, and visualizations into their workspace and immediately start using them.

The workflows can also be downloaded and run on a local Galaxy instance. Because Galaxy workflows are portable, investigators in a large, distributed clinical trial can use the same standardized workflows in multiple locations. Using the same workflows is advantageous both for sharing data and for reproducing analyses. In addition, workflows that have proven successful in previous clinical trials can be widely disseminated and used in the future.

Finally, our workflows can be copied and modified to suit individual analysis needs. Using Galaxy's Web interface, any investigator can edit a workflow, regardless of their programming experience. Potential workflow edits include changing parameter settings and substituting a new tool into a workflow. For example, instead of using VarScan as a variant caller in, another variant caller could be used. As better performing tools become available in Galaxy, we intend to introduce them into our curated pipelines to ensure that our pipelines use robust algorithms.

### Design philosophy

Our pipeline development approach is motivated by a few key principles. We used open-source tools to make our pipelines widely available and transparent. When available, established and/or best practices are used. We designed the pipelines to be modular so that different components could be substituted or added and parameters could be modified. For example, instead of automatically filtering (reducing) variants based on certain criteria such as minor allele frequency, variants are annotated and then an explicit filtering operation is applied. This is very useful within the context of Galaxy because investigators can easily modify workflows, such as by changing variant filtering criteria, using its graphical editor. Modularity ensures that the pipelines can evolve and incorporate new tools as they become available rather than requiring the development of new pipelines.

The exome and transcriptome analysis pipelines require vastly more time and computing resources than the variant analysis pipeline: the exome/transcriptome processing pipelines require about a day to complete on a small computing cluster, while the integrated variant analysis pipeline can be run in less than an hour. Also, there are established protocols for exome and transcriptome processing but less so for variant analysis. Hence, by splitting the pipelines up as we have and putting the pipelines in Galaxy, it is simple and fast to experiment with different settings in the variant analysis pipeline and find settings that are most useful for a particular set of samples.

## Results

### Validation using cell line data

**Figure 2:**
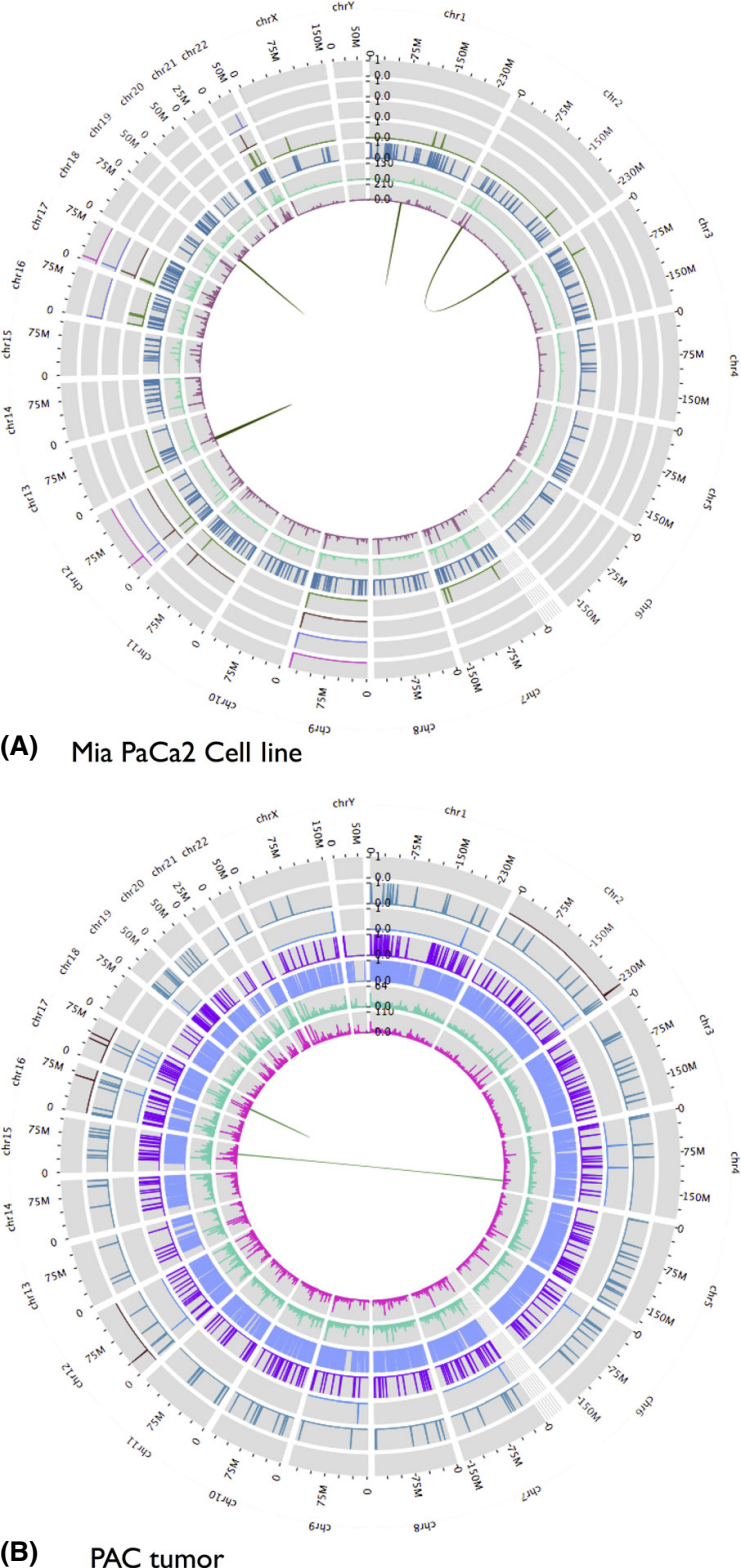
Galaxy Circos plot showing data produced from (A; at top) exome and transcriptome analysis of Mia PaCa2 cell line and (B; at bottom) transcriptome analysis of a pancreatic adenocarcinoma tumor. Starting at the innermost track, the data are: (i) mapped read coverage; (ii) mapped read coverage after PCR duplicates removed; (iii) called variants; (iv) rare and deleterious variants; (v) rare, deleterious, and druggable variants; (vi) rare and deleterious variants in highly expressed genes; (vii) rare, deleterious, and druggable variants in highly expressed genes. Read coverage data shown are for mapped exome reads for cell line and mapped transcriptome reads for tumor.

To validate our pipelines, we analyzed targeted exome and whole transcriptome sequencing data from three well-characterized pancreatic cancer cell lines: MIA PaCa2 (MP), HPAC, and PANC-1. Exonic regions of 577 genes that are commonly included in cancer gene panels were sequenced. All three cell lines are included in the Cancer Cell Line Encyclopedia (CCLE) 15; the CCLE includes a mutational profile for known oncogenes and drug response information for each cell line. The goal of this analysis is to use our pipelines to process the cell line sequence data, compare the output from our pipelines to CCLE entries, and determine whether our pipelines produce results that concur with known findings. Concordance with known findings will validate our pipelines’ performance Figure2A shows an interactive Galaxy-Circos plot of data generated from analysis of the MIA PaCa2 cell line.

Table S1 summarizes the results obtained from the exome pipeline for each cell line's exome sequencing data, and Table S2 summarize results from the transcriptome pipeline for each cell line's transcriptome sequencing data. Between 6200 and 7000 variants were identified in each cell line's targeted exome, and 27-33% of genes in each cell line were expressed at greater than 10 FPKM, the threshold for highly expressed genes in the variant analysis pipeline.

Table1 lists gene fusions found by the transcriptome analysis pipeline and summarizes the results obtained from the variant analysis pipelines for each cell line. The CCLE includes 84 mutations—single-nucleotide polymorphisms (SNPs) and small insertions/deletions—in MP and 2 each for HPAC and PANC-1. 23 MP mutations and all mutations for the other cell lines fall within our targeted exome regions. All CCLE mutations, including SNPs and small insertions and deletions, were found in our cell lines. About 30 rare, deleterious mutations were found in each cell line, with 4-6 found in the COSMIC cancer database for each line. Rare, deleterious, and druggable mutations were reported in many genes associated with cancer, including *ALK, CDKN2A, KRAS*, *NOTCH1, TOP1* (topoisomerase 1), and *TP53*. Reported druggable mutations in highly expressed genes occurred in *CDKN2A, KRAS*, *NOTCH1,* and *TP53*.

**Table 1:** Results obtained using molecular profiling and drug targeting pipeline on three common pancreatic cancer cell lines

	MIA PaCa2	HPAC	PANC-1
Gene fusions	*CRIM1*-*IQCA1*	*IRAK3*-*RBMS1*	None
	*BCAR3-GCLM*		
Variants (ts/tv ratio)	6214 (2.14)	6990 (2.13)	6821 (2.15)
Rare and deleterious (RD) variants	31	31	25
RD variants in COSMIC	6:	6:	4:
	516	521	521
	10656	12479	10660
	28763	132780	28763
	132780	256119	1133963
	256119	1133963	
	431727	1182405	
Genes with RD variants	20	21	18
RD and druggable variants [in COSMIC]	5 [3 in COSMIC]	4 [2]	4 [3]
Druggable genes	5:	4:	4:
	*BCR*	*CDKN2A*	*ALK*
	*BIRC3*	*KCNH2*	*KRAS*
	*KRAS*	*KRAS*	*NOTCH1*
	*NOTCH1*	*TP53*	*TP53*
	*TP53*		
Potential drugs	31 drugs	28	29
RD variants (expression filtered)	8	10	10
RD variants in COSMIC (expression filtered)	3:	3:	3:
	516	521	521
	10656	12479	10660
	28763	1133963	
Genes with RD variants (expression filtered)	5	9	8
RD, and druggable variants [in COSMIC] (expression filtered)	3 [3 in COSMIC]	2 (2)	2 (2)
Druggable genes (expression filtered)	3:	2:	2:
	*KRAS*	*CDKN2A*	*KRAS*
	*NOTCH1*	*KRAS*	*TP53*
	*TP53*		
Potential drugs (expression filtered)	22 drugs	19	18

Molecular profiling includes mutations and gene expression data obtained by analyzing high-throughput sequencing data from targeted exome (577 genes often included in cancer panels) and whole transcriptome sequencing assays. The *ts/tv* metric is the ratio between mutation transitions versus transversions. RD, rare and deleterious.

These mutation results are consistent with the CCLE data. The CCLE includes drug response data for MP, HPAC, and for two cell lines that have a mutational profile similar to PANC-1: KP-1N and KP-1NL. As expected, all cell lines show deleterious mutations in *KRAS* 40,41. Although *KRAS* has long be considered an undruggable target, new strategies that look beyond canonical Ras-Raf-MEK-ERK pathway signaling to target mutant KRAS are promising 42–44. CCLE drug response data indicate that all cell lines appear responsive to MEK inhibitors, which are supported by animal models 45. Curiously, the outputs for *CDKN2A* and *TP53*, known tumor suppressor genes, was contrary to the expectation of loss of function and indication of their druggability highlights the requirement for tertiary filtering of actionable changes by knowledgeable end users.

For MIA PaCa2, rare and deleterious variants were found in five potential druggable genes, with variants in three expressed genes: *KRAS*, *TP53*, and *NOTCH1*. CCLE drug response data for MIA PaCa2 indicate that it is also sensitive to compounds that target MEK. Comparing overall drug response profiles, the KP-1N and KP-1NL cell lines show less response than MIA PaCa2 and HPAC, which agree with the data in Table1 showing fewer known druggable mutations and genes in PANC-1 as compared to MIA PaCa2 and HPAC. Gene fusions were found in MIA PaCa2, and a single fusion was found in HPAC.

### Comparing primary PAC tumors with cell lines

**Figure 3:**
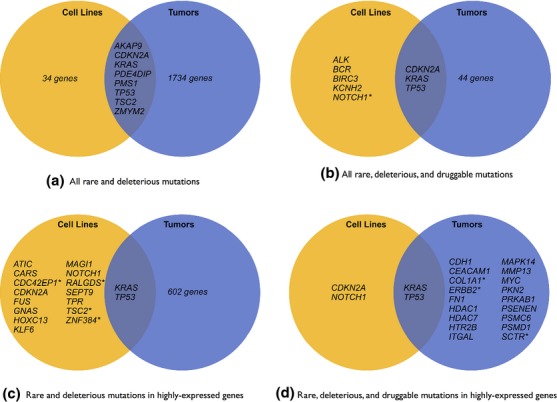
Shared mutations between three pancreatic cancer cell lines—MIA PaCa2, HPAC, and PANC-1—and three primary pancreatic adenocarcinoma (PAC) tumors. From top to bottom, left to right: (A) all shared rare and deleterious mutations; (B) shared rare, deleterious, and druggable mutations; (C) shared rare and deleterious mutations in highly expressed genes; and (D) shared rare, deleterious, and druggable mutations in highly expressed genes. Asterisks (*) indicate that multiple cell lines or tumors showed mutations in the gene. Shared mutations were found in genes specifically associated with PAC (*KRAS*, *PDE4DIP*) and also genes implicated in many cancers (*CDKN2A, TP53*). Potentially druggable mutations in highly expressed genes shared between cell lines and tumors reside in two oncogenes: *KRAS* and *TP53*. Lastly, druggable mutations are significantly more limited in the cell lines as compared to tumor mutations.

We have applied our pipelines to compare six primary PAC tumors with the three cell lines discussed previously. We sequenced six primary PAC tumors using whole transcriptome sequencing. Exome sequencing was not performed for these tumors, which provided an opportunity to use RNA-seq exclusively for characterizing PAC tumors. Figure3 shows data generated from the analysis of one tumor using a Circos plot generated in Galaxy.

Table S2 summarizes results obtained from using the transcriptome pipeline to analyze tumor sequence data, including mapped reads, gene expression, and called variants. Table2 lists gene fusions found and summarizes results obtained from the variant analysis pipelines for each tumor; input to this pipeline was the gene expression and variant datasets produced from the tumor transcriptome pipeline. Figure2B shows an interactive Galaxy-Circos plot of data generated from analysis of tumor 2.

**Table 2:** Results obtained using molecular profiling and drug targeting pipeline on RNA-seq data for six primary pancreatic cancer tumors

	T1	T2	T3	T4	T5	T6
Gene fusions	*KANSL1*-*ARL17A*	*TRIM2*-*ANXA2*	None	None	None	None
		*KANSL1*-*ARL17A*				
Variants (ts/tv ratio)	57,388 (3.02)	119,188 (2.88)	96,212 (2.91)	54,960 (2.88)	77,385 (2.84)	62,532 (2.87)
Rare and deleterious (RD) variants	454	664	665	516	521	372
RD variants in COSMIC	13	15	12	11	8	8
Genes with RD variants	358	522	513	401	395	292
RD and druggable variants [in COSMIC]	4 [0]	15 [2]	16 [2]	11 [0]	10 [1]	6 [0]
Druggable genes	4:	14:	15:	9:	8:	6:
	*CEACAM1*	*CDH1*	*ERBB2*	*CA4*	*ATM*	*F5*
	*HDAC7*	*CDKN2A*	*F8*	*CLCN2*	*CHEK2*	*FGF2*
	*MYC*	*CENPE*	*HCAR2*	*COL1A1*	*CSF2RA*	*NOTCH3*
	*UGCG*	*COL1A1*	*JAK2*	*CSF2RA*	*ITGAL*	*SCNN1D*
		*ERBB2 F8*	*KRAS*	*FN1*	*MAPK14*	*SIRT1*
		*HDAC9*	*MMP13*	*HDAC1*	*SCTR*	*TGFB1*
		*KDR*	*MUC16*	*HTR2B*	*TP53*	
		*KRAS*	*MYC*	*PSENEN*	*XIAP*	
		*MUC16*	*PRKAB1*	*ZHX2*		
		*NOTCH4*	*PSMC6*			
		*PIK3C2B*	*RICTOR*			
		*PRKCA*	*SCNN1D*			
		*PSMD1*	*SCTR*			
			*TP53*			
			*TRPV6*			
Potential drugs	14 drugs	117	65	31	21	9
RD variants (expression filtered)	149	181	196	175	176	89
RD variants in COSMIC (expression filtered)	1	2	5	3	6	2
Genes with RD variants (expression filtered)	126	151	155	141	135	71
RD, and druggable variants (in COSMIC) (expression filtered)	3 (0)	5 (1)	6 (2)	5 (0)	5 (1)	0 (0)
Druggable genes (expression filtered)	3:	5:	6:	5:	4:	0
	*CEACAM*	*CDH1*	*ERBB2*	*COL1A1*	*ITGAL*	
	*HDAC7*	*COL1A1*	*KRAS*	*FN1*	*MAPK14*	
	*MYC*	*ERBB2*	*PRKAB1*	*HDAC1*	*SCTR*	
		*KRAS*	*PSMC6*	*HTR2B*	*TP53*	
		*PSMD1*	*SCTR*	*PSENEN*		
Potential drugs (expression filtered)	13 drugs	42	43	24	6	0

The *ts/tv* metric is the ratio between mutation transitions versus transversions. RD, rare and deleterious.

These results show the challenges inherent in sequencing PAC tumors. PAC tumors are very difficult to biopsy or remove cleanly, and sequenced tumor samples nearly always include significant amounts of stromal (normal) tissue. Sequence data obtained from a mixed population of tumor and stromal cells often masks signals, and we found this to be true for our tumors as well. Although the majority of PAC tumors show *KRAS* mutations 46, we found *KRAS* mutations in only two tumors analyzed. This appeared to be due to lack of read coverage for *KRAS* in the other tumors sequenced, and we asked for re-evaluation of the tumor sections by a certified pathologist. This re-review by a pathologist identified very low tumor percentage in two of the four *KRAS* wild-type samples that we sequenced, which emphasizes the invaluable caveat of appropriate sample quality control assessment by a trained pathologist prior to nucleic acid preparation and sequencing 47. Finally, false positives for mutations in homologous genes are common when using RNA-seq because mapping spliced reads is difficult. However, the stringent variant filtering in our pipeline is designed to effectively remove the great majority of these false positives.

Comparing mutations between cell lines and sequenced PAC tumors (Fig.3) shows important similarities and differences. All three cell lines and two tumors share *KRAS* mutations, with the HPAC and PANC-1 cell lines and two tumors sharing the exact mutation (COSMIC521). This observation aligns with general consensus about the importance of the *KRAS* pathway in PAC. Tumors and cell lines also share rare, deleterious mutations in the following genes: *AKAP9*, *CDKN2A*, *PDE4DIP*, *PMS1*, *TP53*, *TSC2*, and *ZMYM2*. *TP53* mutations appeared in two cell lines and three tumors, and rare and deleterious mutations in *PDE4DIP* were most prevalent, appearing in all tumors and MIA PaCa2 and PANC-1 cell lines. A recent whole-genome sequencing study of PAC also found evidence of mutations in *PDE4DIP* 48, which has been reported as being highly expressed in esophageal squamous cell carcinoma 49. Also, an intronic SNP (rs2863344) in *PDE4DIP* has been associated with response to capecitabine 50, a common therapy for gastric and breast cancers, which indicates that expression and mutation status of this gene may be relevant to an informed treatment strategy for pancreatic cancer.

Mutations in the genes *COL1A1*, *ERBB2* (aka *HER2)*, and *SCTR* were found in two tumors each but not in any cell lines. *ERBB2* mutations may be of particular interest because the two tumors that include *KRAS* mutations also include *ERBB2* mutations, and *KRAS* and *ERBB2* are thought to function jointly to drive tumor growth 51. Because *ERBB2* is a druggable target 52, cell lines that exhibit only *KRAS* but not *ERBB2* mutations may not be appropriate models for PAC tumors with both *KRAS* and *ERBB2* mutations. Two tumors also show evidence of a *KANSL1*-*ARL17A* gene fusion, but there is no evidence of this fusion in the cell lines. Other gene fusions in cancer cell lines include *ARL17A* 32, so the presence of a *KANSL1*-*ARL17A* in PAC tumors may warrant additional investigation.

### Performance and scalability

We analyzed the tumor cell line sequence data using the public Galaxy server (http://usegalaxy.org). To ensure privacy of patient sequence data, patient tumor data were analyzed using Galaxy installed on a local computing cluster. Galaxy integrates well with many different high-performance computing clusters and can scale to use all available computing resources to process very large tumor sequencing datasets. Given high-performance computing resources, then, Galaxy and our pipelines can analyze arbitrarily large tumor sequence data sets.

The main public Galaxy instance (http://usegalaxy.org) runs jobs on TACC, which is part of the XSEDE national high-performance computing environment (https://www.xsede.org). The largest cell line data set is the PANC-1 exome sequence, which includes ∽151 million 100bp paired-end reads or ∽30 billion bases. The exome analysis pipeline is perhaps the most time-intensive pipeline with three long processes—read alignment, duplicate removal, and variant calling. On the main public server, this pipeline ran in ∽36 h, although 50% of this time was spent waiting for computing resources to become available. Thus, compute time for exome analysis pipeline was ∽18 h. The largest RNA-seq data set came from Mia PaCa2, which contains ∽31 million 100bp paired-end reads or ∽6 billion bases, and the transcriptome analysis pipeline ran in ∽24 h, including waiting time. Compute time for the transcriptome pipeline, then, is ∽12 h. The integrated variant analysis pipeline runs in ∽15 min for all cell line data sets and has no waiting time because analysis steps are not compute intensive.

Patient tumor RNA-seq data are smaller than the cell line RNA-seq data, averaging ∽28 million 100bp paired-end reads or ∽5.6 billion bases. On two dedicated compute nodes, each with 24 compute processors, the transcriptome analysis pipeline and variant calling completed in ∽18 h and the integrated variant analysis ran in ∽15 min.

## Discussion

Using Galaxy as a platform for cancer genome analysis pipelines has important advantages for translational cancer research and applications. Galaxy pipelines provide completely specified analyses that can be used as standardized analysis protocols to generate uniform data. Standardized analyses and uniform data can improve clinical studies by making it possible to do reproducible analyses across different sites and to share and aggregate data.

Galaxy also provides other features necessary for doing high-quality cancer genome clinical studies. Galaxy can serve as an analysis hub for clinical studies, which often include a mixture of personnel, only some of which have programming expertize. Bioinformaticians can automate analyses using Galaxy's API, while investigators without programming knowledge can use Galaxy's Web interface to view and run pipelines using only a small number of mouse clicks. Galaxy, then, makes analysis tools and workflows available to all personnel in a clinical study.

Galaxy pipelines are modular so that investigators can update pipelines as new tools become available; however, pipelines are also versioned so that previous iterations are saved and recoverable. Finally, Galaxy provides infrastructure for visualizing, reproducing, and sharing analyses, all of which are essential for clinical studies.

Despite the value of these computational tools, this investigation also highlights the challenges in interpreting and using tumor genomic features to guide treatment. Our pipelines identified *KRAS* and *TP53* as potentially druggable targets for both the cell lines and tumors, which can be misleading, particularly in a clinical context. This emphasizes the critical need to have knowledgeable end users to interpret the data as well as the necessity for more robust and comprehensive druggable mutation databases. However, we are confident that this tool can be used to find actionable targets as well as identify the most appropriate cell lines, particularly those that are nontraditional research models, to be used as preclinical models that more closely match tumor genotypes. In fact, using CCLE data with our tumor data and this analysis pipeline (data not shown), it appeared that the less common KP-1N and KP-1NL cells, may be better preclinical models for the tumors that we tested than the more commonly used MIA PaCa2, HPAC, and PANC-1 cell lines.

## Conclusions

There are many computational challenges that arise when developing translational cancer genomics applications, particularly those with the goal of personalized oncology. Multi-tool pipelines are required to analyze and integrate different types of -omic data and to combine private patient data with public databases. These pipelines require appropriate securities to protect patient privacy and need to be accessible to investigators without programming experience. Finally, they should be completely reproducible and transparent so that the pipelines can be used for standardized analysis and data produced from the pipelines can be readily compared across clinical settings.

The pipelines discussed in this paper are a first attempt to meet these criteria for tumor variant analysis. Our pipelines provide end-to-end support for analyzing variants from high-throughput exome and transcriptome tumor sequencing. The exome analysis pipeline call variants, and the transcriptome analysis pipeline call variants, computes gene expression, and identifies fusion genes. The variant analysis pipeline annotates and filters variants to identify rare, deleterious variants that are likely associated with disease, and further provides lists of rare, deleterious variants in expressed genes as well as those that are druggable. These pipelines are made widely accessible and reproducible via their integration with Galaxy. Galaxy also provides useful visualization and sharing features for pipelines and produced data.

We used these pipelines to analyze sequence data from six PAC tumors and three common cell lines. We validated previously published mutational and drug response data for the cell lines. Our analysis of the tumors showed that they shared common *KRAS* mutations with the cell lines. However, the tumors also exhibited *ERBB2* mutations not found in the MIA PaCa2, HPAC, and PANC-1 cell lines, indicating the need to re-evaluate preclinical models of therapeutic response in the context of genomic medicine.

## Methods

### Cell Line and tumor tissue acquisition and processing

The MIA PaCa2, HPAC, and PANC-1 cell lines were obtained as frozen aliquots from ATCC (http://www.atcc.org/). A total of six de-identified pancreatic tumor frozen specimens were available for this study through an IRB approved tissue banking protocol. Genomic DNA and total RNA were isolated using Omega BioTek (http://www.omegabiotek.com/) chemistries according to the manufacturer's protocols. DNA was quantitated using NanoDrop and Qubit, and RNA was quantitated using NanoDrop and Agilent BioAnalyzer.

### Library preparation and sequencing

Total RNA from pancreatic cell lines and tumor tissue all had RIN>8.0 and were prepared using the Illumina TruSeq RNA kit (v1) according to manufacture's protocols. Final RNA-Seq libraries were quantitated using qPCR and Agilent BioAnalyzer and sequenced using 100 bp paired-end reads at 100,000 reads per sample with an Illumina HiSeq 2000 instrument.

Custom cancer exome sequencing (WES) was performed using genomic DNA prepared from the three pancreatic cell lines. Libraries were prepared using a 577 gene cancer exome panel designed and run in duplicate using Agilent SureSelect and NimbleGen SeqCapEZ library preparation methods. All three cell lines were run as SureSelect and SeqCapEZ libraries, for a total of six libraries that were sequenced in a single lane of a 100 bp paired-end run on a HiSeq 2000.

FASTQ file generation and initial data QC were performed using a CASAVA v1.8.1 software (Illumina) for both the RNA-Seq and cancer exome data sets. Uniformity of coverage and overall data quality for the cancer exomes was consistent with what has been reported previously for Agilent SureSelect and NimbleGen SeqCapEZ whole exome sequencing kits 52. FASTQ files were used as the input data for the Galaxy analysis pipeline. Cell line exome and transcriptome sequencing data is available in two places: (a) in the NCBI SRA under accessible numbers SRX472933 and SRX472980 (Mia PaCa2), SRX472944 and SRX473000 (HPAC), and SRX472948 and SRX473014 (PANC-1); and (b) in the main public Galaxy instance at http://usegalaxy.org in a data library named ‘Cancer Cell Lines.’

## Supporting Information

**Table S1.** Analysis results from targeted exome sequencing of cancer cell lines. Targeted exome sequenced 577 genes commonly found in cancer panels. **Table S2.** Analysis results from whole transcriptome sequencing (RNA-seq) of cancer cell lines. **Table S3.** Analysis results from whole transcriptome sequencing (RNA-seq) from six primary pancreatic cancer tumors. Variant analysis and expression analyses were performed.
