# Supplementary material for: Open pipelines for integrated tumor genome profiles reveal differences between pancreatic cancer tumors and cell lines
Source: Cancer Med. 2015 Jan 4;4(3):392–403. doi: 10.1002/cam4.360 (PMC4380965; doi:10.1002/cam4.360)
Supplement: Supplementary file 1 [file cam40004-0392-sd1.docx]

Supplementary Materials

**Sequencing Details**

For targeted exome capture in the cell lines, we used the NimbleGen SeqCap kit to capture the exonic regions of 577 genes that are commonly included in cancer gene panels. Samples were sequenced using a small-insert (~220 base pairs per fragment), paired-end read protocol on an Illumina HiSeq 2000 sequencer. To perform whole transcriptome sequencing of the cell lines and tumors, we captured mRNA and sequenced using a small-insert (~220 base pairs per fragment), paired-end read protocol on an Illumina HiSeq 2000 sequencer.

**Targeted Exome and Transcriptome Analysis Results**

These are the results obtained using our pipelines to analyze cell line targeted exome and whole transcriptome high-throughput sequencing data.

|  | **MIA PaCa2** | **HPAC** | **PANC-1** |
| --- | --- | --- | --- |
| **Raw Reads** | 66,120,403 | 65,226,188 | 75,562,531 |
| **Mapped Reads** | 64,150,060 (97.0%, 98% aligned in pairs) | 63,111,849 (96.8%, 98.5% aligned in pairs) | 73,183,252 (96.7%, 98.1% aligned in pairs) |
| **Mapped Reads, No duplicates** | 34,670,477 (54.0% of aligned reads, 52.4% overall) | 31,675,365 (50.2% of aligned reads, 48.6% overall) | 29,277,483 (40.0% of aligned reads, 38.7% overall) |
| **Variants in Target Regions, Allele Frequency >= 10%** | 6,214 | 6,990 | 6,821 |

**Table S1**. Analysis results from targeted exome sequencing of cancer cell lines. Targeted exome sequenced 577 genes commonly found in cancer panels.

|  | **MIA PaCa2** | **HPAC** | **PANC-1** |
| --- | --- | --- | --- |
| **Raw Reads** | 31,427,876 | 24,399,702 | 17,669,439 |
| **Mapped Reads** | 28,748,144 (91.4%, 67.6% mapped in pairs) | 22,333,284 (91.5%, 66.5% mapped in pairs) | 16289963 (92.2%, 68.3 mapped in pairs) |
| **Genes with FPKM > 0 / 10 / 100** | 15,382 / 5,091 / 439 | 14,931 / 4,839 / 399 | 23,184 / 6,385 / 632 |

**Table S2**. Analysis results from whole transcriptome sequencing (RNA-seq) of cancer cell lines.

|  | **T1** | **T2** | **T3** | **T4** | **T5** | **T6** |
| --- | --- | --- | --- | --- | --- | --- |
| **Raw Read Pairs** | 21,625,471 | 27,994,737 | 29,300,004 | 21,275,705 | 15,935,179 | 26,514,603 |
| **Mapped Pairs** | 18,821,006 (87.0% overall, 86.2% concordant) | 24,270,353 (86.7% overall, 86.1% concordant) | 24,138,103  (82.4% overall, 81.9% concordant) | 17,546,638 (82.5% overall, 81.5% concordant) | 13,967,634 (87.7% overall, 86.5% concordant) | 21,990,863 (82.9% overall, 82.5 concordant) |
| **Mapped Reads, No duplicates** | 9,548,349 (50.7% of mapped, 44.2% overall) | 17,547,503 (72% of mapped, 62.7% overall) | 15,469,745 (64.1% of mapped, 52.8% overall) | 9,934,800 (56.6% of mapped, 46.7% overall) | 11,010,983 (78.8% of mapped, 69.1% overall) | 6,765,345 (30.8% of mapped, 25.5% overall) |
| **Variants with Allele Frequency >= 30% (% of total called)** | 57,388 (50.3%) | 119,188 (51.0%) | 96,212 (47.4%) | 54,960 (48.6%) | 77,385 (58.3%) | 29,399 (47.0%) |
| **Genes with FPKM >(0, 10, 100)** | 17,110 / 4590 / 327 | 18,060 / 5243 / 402 | 18,037 / 5,560 / 512 | 17,578 / 4,797 / 409 | 18,042 / 5726 / 368 | 17,119 / 4017 / 342 |

**Table S3**. Analysis results from whole transcriptome sequencing (RNA-seq) from six primary pancreatic cancer tumors. Variant analysis and expression analyses were performed.
